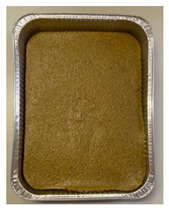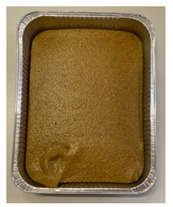# Tartary Buckwheat: A New Plant-Based Ingredient to Enrich Corn-Based Gluten-Free Formulations

**DOI:** 10.3390/foods10112613

**Published:** 2021-10-28

**Authors:** Marta Appiani, Noemi Sofia Rabitti, Cristina Proserpio, Ella Pagliarini, Monica Laureati

**Affiliations:** Sensory & Consumer Science Lab (SCS_Lab), Department of Food, Environmental and Nutritional Sciences (DeFENS), University of Milan, 20133 Milan, Italy; noemi.rabitti@unimi.it (N.S.R.); cristina.proserpio@unimi.it (C.P.); ella.pagliarini@unimi.it (E.P.); monica.laureati@unimi.it (M.L.)

**Keywords:** tartary buckwheat, liking, hedonic response, consumers, food acceptability, just-about-right

## Abstract

Tartary buckwheat is a pseudocereal receiving increasing attention as a minor crop interesting for agrobiodiversity conservation and sustainability. It is rich in bioactive substances which, however, may lead to sensory properties undesirable to the consumer, such as bitterness and astringency. The aim was to evaluate consumers’ perception and overall liking of food products enriched with tartary or common buckwheat. A total of 120 consumers (56% women) aged 20–60 years (mean age ± SD: 38.8 ± 13.0 years) evaluated six samples of a corn-based gluten-free formulation enriched by increasing concentrations (20%, 30%, 40%) of either common (CB) or tartary buckwheat (TB) flour for overall liking and appropriateness of sensory properties. Results showed significant differences (*p* < 0.0001) in liking among samples. Considering all subjects, liking decreased with the increase of tartary buckwheat additions, although TB20 and TB30 samples were well accepted and comparable to all CB samples. TB40 was the least liked product. Two clusters of consumers showing opposite behaviours according to liking were found. One cluster (30%) showed an increased liking with the increasing amount of tartary buckwheat. These results show that by keeping the concentration of tartary buckwheat up to 30%, it is possible to develop new products accepted by consumers.

## 1. Introduction

Current food systems are no longer sustainable. Food production, contributing to climate change, freshwater use, and biodiversity loss, is a major driver of global environmental change [1]. For this reason, it is necessary to implement a sustainable transformation at the level of the entire food chain to ensure healthy food systems. A sustainable food system must also aim for sustainable agricultural production, which should become increasingly focused on preserving biodiversity and providing essential nutrients for balanced diets rather than increasing the volume of a few crops.

Biodiversity for food and agriculture is essential to ensure food security and is directly associated with dietary health. Sustainable healthy diets include an abundance of plant-based foods focused on seasonal and local products, such as wholegrains, legumes, nuts, and fruit and vegetables [2]. It is estimated that more than 75% of the genetic diversity of agricultural crops worldwide has been lost [3]. In this context, the exploitation and use of minor crops are very important to increase environmental sustainability since they require only limited inputs. Furthermore, local crops could be incorporated into food recipes as functional ingredients that are able to enhance the nutritional profile of foods and their potential pro-health effects.

In recent years, among minor crops, the attention for tartary buckwheat (*Fagopyrum tataricum* Gaertn.), a pseudo-cereal belonging to the *Polygonaceae* family, has increased considerably. It likely originated in northwestern Yunnan in China [4]. Nowadays, it is cultivated in the mountainous areas of China, India, Bhutan, and Nepal. In Europe, it grows in some regions of Slovenia, Italy, and Northern Europe [5]. This crop, unlike common buckwheat (*Fagopyrum esculentum* Moench.), is resistant to high-altitude environmental conditions and is able to grow even in marginal growing areas with minimal agricultural infrastructure that are characterised by a dry, thin soil poor in nutrients [6] and a low temperature climate [7]. Until a few years ago, this pseudocereal was considered a weed in common buckwheat crop [6,8]. However, in recent years, it is receiving increasing interest as an ingredient for the development of new functional foods [5], being rich in dietary beneficial components. It is also gluten-free and therefore suitable for people with celiac disease [9].

The tartary buckwheat flour contains proteins with high biological value and balanced amino acid composition, in particular lysine, isoleucine, tryptophan, valine, histidine, and phenylalanine [10]. The protein content is higher (10%) in comparison with other commonly used cereals [11]. This pseudocereal also has a good fatty acid composition (2%) consisting mainly of unsaturated fatty acids (74.5%) compared to saturated ones (25.3%) [8].

Furthermore, tartary buckwheat flour represents a good source of dietary fibre [12], the intake of which is below the recommended daily amount (from 25 to 38 g/day) for most individuals [13]. In particular, Bonafaccia et al. [8] have shown that the total dietary fibre (TDF) content of tartary buckwheat flour is 6.29%, with that of soluble and insoluble fibres being 0.52% and 5.77%, respectively. This pseudocereal is also characterised by the presence of resistant starch, which is considered a type of dietary fibre since it is the fraction of starch that resists digestion by not being hydrolysed in the small intestine but is fermented by the gut microflora in the colon. Qin et al. [14] have shown that the resistant starch content in the flour of tartary buckwheat cultivars ranges from 13.06% to 22.53%.

Moreover, this pseudocereal is a richer source of nutritionally important minerals than many common cereals, containing high levels of zinc (Zn), copper (Cu), manganese (Mn), potassium (K), and magnesium (Mg) [10]. A further reason why tartary buckwheat is an excellent food ingredient is its vitamin profile [8], consisting of a higher vitamin B group content, especially vitamin B1 (thiamine), B2 (riboflavin), B3 (niacin), and B6 (pyridoxine), than common buckwheat [11].

Among buckwheat species, tartary buckwheat is particularly distinguished by its high amounts of rutin (flavonol 3-*O*-rutinoside) and other polyphenols [6,15,16,17] located in its seeds, leaves, flowers, and cotyledons [18]. Kitabayashi et al. [19] found that the content of rutin in tartary buckwheat is almost 100 times higher than that of common buckwheat. Rutin is a bioflavonoid with antioxidant [20] and anti-inflammatory activity [21]. Specifically, rutin promotes the health and elasticity of blood vessels by improving blood circulation and preventing bleeding [22], prevents high blood pressure, and reduces the glycemic index. Tartary buckwheat seeds also contain high levels of rutinosidase activity (F3g) [23,24], a ß-glycosidase that hydrolyses rutin to quercetin and the disaccharide rutinose immediately after the addition of water in the prepared flour [25,26]. Despite the high nutritional value of tartary buckwheat, currently this pseudocereal is infrequently used in food products because of its bitter taste. In fact, the rutinosidase activity, in addition to reducing the rutin content, is responsible for the bitter taste of tartary buckwheat. It has been shown that the bitter compounds in tartary buckwheat are quercetin and additional unidentified compounds [26,27]. Quercetin has specific activity on the bitter taste receptor TAS2R14 [28]. The higher content of these bitter compounds in tartary buckwheat might be one of the reasons why the consumption of common buckwheat is currently much more widespread.

The development of food products with new functional ingredients is a real challenge for the food industry, as nutritional and sensory improvements have to be balanced. Perceptual factors, such as taste preferences and perceived sensory properties (the taste, smell, texture, and appearance of a food) are among the main barriers influencing consumers’ choice of healthy and sustainable foods [29,30,31]. Bitter food tends to be rejected by a large portion of consumers [32], making it difficult to develop new food products characterised by this taste stimulus. Moreover, oral sensitivity to sensory stimuli varies greatly among individuals. The most studied phenotypic marker of genetic variability in the perception of bitter taste is the responsiveness to the 6-n-propylthiouracil (PROP status). Individuals can be classified into three PROP taster groups: non-tasters, medium tasters, and super-tasters, which are also reported to differ in eating behaviour and food preferences [33]. In addition to perceptual factors, there are also psychological factors that influence preferences and choices of healthy food, such as food neophobia, which is the reluctance to eat novel or unknown foods [34]. In particular, food neophobia has a negative impact on certain categories of foods, including fruits and vegetables [35,36], characterised by a high intensity of warning sensations, such as bitterness, sourness, astringency [37], and high-fibre products [38].

To the best of our knowledge, no studies have investigated consumers’ acceptance of tartary buckwheat. Therefore, the first objective of the present study was to evaluate consumers’ perception (taste/flavour, appearance, texture, and mouthfeel) and overall liking of food products enriched with either tartary or common buckwheat, in order to identify drivers of liking and rejection of these products. As the food model, a gluten-free food formulation based on corn and buckwheat flour was chosen, which is a typical and largely known product of Northern Italy called “polenta taragna”. Moreover, this type of product is characterised by a very simple matrix, which makes it particularly suitable for pilot productions facilitating its standardisation during preparation. A second objective was to investigate whether there are clusters of consumers who are more willing to accept tartary buckwheat food products and to characterise these clusters according to a series of background variables (i.e., demographics, food neophobia, and responsiveness to PROP) in order to identify potential drivers of liking and rejection of these products.

## 2. Materials and Methods

### 2.1. Participants

A total of 120 volunteers (67 women and 53 men) aged 20–60 years (mean age ± SD: 38.8 ± 13.0 years) were recruited among students and employees of the Faculty of Agriculture and Food Sciences of the University of Milan and by a professional sensory and market research agency. Subjects were divided into three age groups: 18–30 years old (35%), 31–45 years old (32%), and 46–60 years old (33%). Only subjects who were ≥18 years of age, who did not suffer from food allergies or intolerances and who liked gluten-free food formulations based on corn and buckwheat flour, were involved in the study.

All subjects gave their written informed consent prior to the beginning of the study, and they were instructed to refrain from smoking, eating, and drinking (except water) in the hour before tasting. The present study was performed according to the principles established by the Declaration of Helsinki [39] and the protocol was approved by the Ethical Committee of the University of Milan (n. 11/21).

### 2.2. Food Samples

Six gluten-free samples of a corn-based formulation were produced, enriched by the addition of increasing concentrations of common buckwheat (CB) flour (Raetia Biodiversità Alpine, Teglio, Sondrio, Italy) or tartary buckwheat (TB) (Raetia Biodiversità Alpine, Teglio, Sondrio, Italy). Common buckwheat (CB) flour or tartary buckwheat (TB) flour were added at various concentrations: 20% (CB20; TB20), 30% (CB30; TB30), and 40% (CB40; TB40) to corn flour (Molino Filippini S.r.l., Teglio, Sondrio, Italy) (Table 1). The addition levels were defined based on the percentages of addition most frequently used in similar commercial products and according to preliminary trials performed by a group of six expert assessors with the aim of evaluating if the different additions of common or tartary buckwheat allowed products distinguishable from each other in terms of sensory properties (e.g., bitterness and overall flavours) to be obtained, while keeping a good compromise with liking.

Food formulations were made by cooking 400 g of flour (corn flour + either common buckwheat or tartary buckwheat) in 1.5 kg of water in which 8 g of salt were added. To standardise the preparation of food samples, an automatic cooking-mixer (Thermomix TM 31—Vorwerk Contempora S.r.l., Milano, Italy) was used. Food formulations were prepared by bringing salted water to a boil, adding the flour mix, and cooking the formulation for 40 min at 100 °C.

Samples were prepared the day before the evaluations and, after reaching room temperature, stored at 4 °C. On the day of evaluations, samples were removed from the fridge two hours before the tasting session and cut into 8 cm^3^ cubes of approximately 15 g each which were served at room temperature (approx. 20 °C) in small cups coded with three-digit numbers. Samples were presented in a randomised and balanced order [40].

### 2.3. Experimental Procedure

The evaluations were carried out at the Sensory & Consumer Science Laboratory (SCS_Lab) of the University of Milan and at the laboratory of a sensory and market research agency, following the same experimental procedure. All participants performed the evaluation, which lasted about 30 min, in individual booths under white light.

Firstly, participants completed a short questionnaire to obtain sociodemographic information (age and gender) followed by two questions about familiarity with “polenta” (corn-based formulations) and “polenta taragna” (corn-based formulations with the addition of buckwheat) by using a 5-point labelled scale [41]: 1 = “I do not know it”; 2 = “I know it, but I have never tasted it”; 3 = “I have tasted it, but I don’t eat it”; 4 = “I occasionally eat it”; and 5 = “I regularly eat it”.

Subsequently, participants completed four different tasks: (1) overall liking evaluation of the six samples; (2) food neophobia scale (FNS) assessment; (3) appropriateness of a series of sensory characteristics of the six samples using the just-about-right (JAR) scale; and (4) measurement of PROP responsiveness.

#### 2.3.1. Overall Liking Evaluation

Participants were asked to taste the samples and to express their liking using a 10 cm linear scale anchored by the extremes “Extremely disliked” (rated 0) and “Extremely liked” (rated 100) [40]. The experimenters provided instructions for the evaluation to the participants prior to the tasting. The instructions were “Taste at least half of the sample and wait 10 s before proceeding with the liking evaluation”. Between sample evaluations, participants were asked to rinse their mouths with still mineral water (Levissima S.p.A.) for 15 s.

#### 2.3.2. Food Neophobia Assessment

Food neophobia was measured using the food neophobia scale (FNS) [34], validated in Italian as described by Laureati et al. [37]. The questionnaire consists of 10 items, 5 related to neophilic and 5 related to neophobic attitudes. Participants scored them showing the degree of agreement using a 7-point scale ranging from 1 = “strongly disagree” to 7 = “strongly agree”.

The answers to the 10 items of the FNS were summed up (after reversing the scores of the neophilic items) to have a food neophobia score ranging from 10 to 70. High scores indicate a high level of food neophobia.

#### 2.3.3. Just-about-Right (JAR) Evaluation

After the food neophobia assessment, participants received six newly coded samples and were asked to evaluate how appropriate they considered the level of colour intensity (ochre/yellow colour), bitterness, firmness, dryness, and overall flavour intensity. Five-point just-about-right (JAR) scales were used, anchored to the three following anchors: “Much Too Weak” on the left end, “Just About Right” at the centre, and “Much Too Strong” on the right end [40].

Prior to the assessment, consumers were briefed on how to conduct the assessment and were provided with a description of the attributes. Before tasting the sample, they were asked to evaluate the appropriateness of colour intensity. Then, participants were asked to taste at least half of the sample and to wait 10 s before proceeding with the evaluation of the other selected attributes. Between sample evaluations, they were asked to rinse their mouths with still mineral water (Levissima S.p.A.) for 15 s.

#### 2.3.4. PROP Responsiveness

PROP responsiveness was measured using PROP-impregnated filter paper according to the procedure described by Bartoshuk et al. [42]. Filter papers (4 cm^2^) (Whatman; Cytiva) were soaked in a supra-threshold 3.2 mM solution of PROP. PROP solution (0.5447 g/L) was prepared by dissolving 6-n-propyl-2-thiouracil (European Pharmacopoeia Reference Standard, Sigma-Aldrich, Milano, IT) into deionised water. Papers were air dried and stored at room temperature. The intensity of bitterness was rated using the generalised labelled magnitude scale (gLMS [43]). The gLMS consisted of a 100-unit vertical line with labels placed at “no sensation” (0, bottom of the scale), “barely detectable” (1.4), “weak” (6.1), “moderate” (17.2), “strong” (35.4), “very strong” (53.3), and “the strongest imaginable sensation” (100, top of the scale).

Prior to the test, participants were extensively instructed on the use of the gLMS following published standard procedures [43].

Subjects were asked to consider the ‘‘the strongest imaginable sensation” as the most intense sensation they could ever imagine experiencing considering different sensory modalities (oral and non-oral sensations). In order to become familiarised with the scale anchors, participants were asked to recall remembered sensations [44] such as the cold of a cube of ice in the mouth or the noise of a plane that is flying low. In order to ascertain that the subjects correctly understood how to use the scale, they were asked to rate the light intensity of a camera’s flash on a paper ballot. The criterion to conclude that the subjects understood the use of the scale was that their ratings were higher than “very strong” and lower than “the strongest imaginable sensation of any kind”.

Then, participants were presented with two identical filter cards coded with three-digit numbers and were instructed to place each one on the tongue for 10 s and rate the bitterness intensity using the gLMS. A 60 s break was given to the subjects to rinse their mouths with mineral water after the first evaluation. The average bitterness score was used for each subject.

### 2.4. Data Analysis

The SAS/STAT statistical software package version 9.4 (SAS Institute Inc., Cary, NC, USA), XLSTAT (version 2021.2.1, Addinsoft, Boston, MA, USA), and the Unscrambler X software (CAMO Software AS, Oslo, Norway) were used for the data analysis. Effects showing a *p*-value of 0.05 or lower were considered significant.

#### 2.4.1. Overall Liking Scores

The liking score distribution for each sample was calculated and checked for normality. According to the Shapiro–Wilk test, the distribution was normal for CB20, CB40, and TB30 samples, while the distribution for the CB30, TB20, and TB40 samples deviated from the normal distribution. However, inspection of the Q–Q plots suggested a normal pattern, and thereby all data were handled as normally distributed [45]. Liking of the six samples was analysed by means of the generalised linear model (GLM) considering samples (CB20, CB30, CB40, TB20, TB30, TB40), gender (women and men), and age groups (18–30 years, 31–45 years, 46–60 years) and their second-order interactions as factors. Least-squares means (LS-means) and relevant standard errors (SEM) were computed for each factor. When the GLM showed a significant effect (*p* ≤ 0.05), the Bonferroni test adjusted for multiple comparison was used for post-hoc analyses.

GLM analysis was also used to determine the effect of gender (women and men) and age (18–30, 31–45, 46–60) and their interaction on familiarity with corn-based formulations.

In order to explore clusters of consumers varying in samples liking, a principal component analysis (PCA) was performed on the liking data of the six samples. Considering the subjects’ positions on the map, consumer clusters were then identified through visual interpretation of the loading plot [45]. The differences in liking between the two clusters were studied by means of GLM followed by Bonferroni post-hoc test considering clusters (Cluster 1; Cluster 2), samples (CB20, CB30, CB40, TB20, TB30, TB40), and their interaction as factors. Differences related to gender within clusters were analysed using the chi-square test. Differences within clusters related to age (as continuous variable), familiarity with corn-based formulations and corn-based formulations with the addition of buckwheat, responsiveness to PROP, and food neophobia were analysed using an unpaired *t*-test.

#### 2.4.2. JAR Data

JAR data were combined with overall liking scores and analysed using penalty analysis. Penalty analysis was performed using partial least squares regression [46] to estimate the impact of each attribute of being “much too weak” or “much too strong” on overall liking. According to Plaehn [47] and Ares et al. [46], for each attribute two dummy variables were created to indicate if it was perceived as “much too weak” or “much too strong”. For each attribute, consumer, and sample, “much too strong” intensity was set to 1 if the JAR score was 4 or 5, and to 0 in any other case. In the same way, “much too weak” intensity was set to 1 if the JAR score was 1 or 2 and to 0 in any other case. When the attribute was scored 3, meaning just-about-right (JAR), both dummy variables were set as 0. Therefore, from JAR data, five pairs of dummy variables for the five original JAR variables were created. The PLS regression model was run considering overall liking scores as the dependent variable and the dummy variables as regressors. Full cross validation was chosen as the validation method. In order to explore the “direction” of the relationship between the predictors and response, the regression coefficients were calculated both on the total sample of consumers (*n* = 120) and on the two separate clusters.

## 3. Results

### 3.1. Overall Liking Assessment

**Figure 1:**
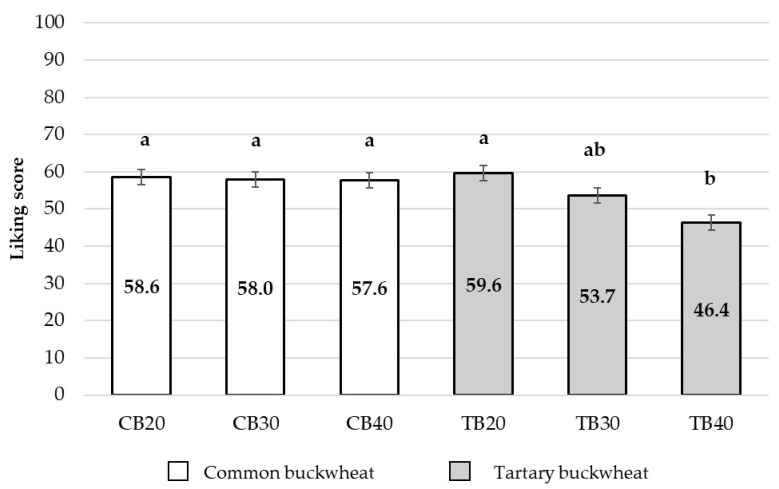
LS-mean liking score obtained for samples (CB20, CB30, CB40, TB20, TB30, TB40). Error bars represent standard error of mean (SEM). Different letters within graph indicate significant differences according to Bonferroni post hoc test (CB = common buckwheat; TB = tartary buckwheat).

The mean liking scores by samples are provided in Figure 1. The main factor “*Samples*” was found to have a significant effect on liking (F_(5,694)_ = 6.40, *p* < 0.0001). Sample TB20 obtained the highest score (LS-mean = 59.6; SEM = 2.0) and was equally preferred to samples CB20, CB30, and CB40 (CB20: LS-mean = 58.6; SEM = 2.0; CB30: LS-mean = 58.0; SEM = 2.0; CB40: LS-mean = 57.6; SEM = 2.0) and to sample TB30 (LS-mean = 53.7; SEM = 2.0). Overall, the samples were well accepted, with mean liking scores above the middle of the scale (range 53.7–59.5), with the exception of TB40, which received the lowest liking score (LS-mean = 46.4; SEM = 2.0). The addition of increasing concentrations of tartary buckwheat produced a decrease in samples liking, while this trend was not observed for samples with common buckwheat addition.

No interaction was significant. A significant “*Age groups*” effect on liking scores was found (F_(2,694)_ = 12.71; *p* < 0.0001). Consumers aged 46 to 60 years displayed a higher degree of liking (LS-mean = 61.4; SEM = 1.4) compared to those aged 18 to 30 (LS-mean = 52.9; SEM = 1.4) and 31 to 45 (LS-mean = 52.6; SEM = 1.4), which did not significantly differ from each other. No significant “*Gender*” effect was found on liking scores (*p* = 0.95), indicating that samples were equally liked by both men and women.

### 3.2. Effect of Gender and Age on Familiarity

The age gender interaction was not significant for corn-based formulation (*p* = 0.17), whereas it was for corn-based formulation enriched with buckwheat (F_(2,719)_ = 3.8; *p* = 0.02). No gender-related differences in familiarity with corn-based formulation and corn-based formulation with the addition of buckwheat were found (*p* = 0.97 and *p* = 0.73, respectively), while age-related effects were found for both food formulations (F_(2,719)_ = 46.6, *p* < 0.0001; F_(2,719)_ = 36.2, *p* < 0.0001, respectively). For younger consumers (18–30 years), corn-based (LS-mean = 4.3; SEM = 0.03) and corn-based formulation with buckwheat (LS-mean = 4.1; SEM = 0.04) were significantly less familiar than for consumers aged 31 to 45 (LS-mean = 4.8; SEM = 0.03; LS-mean = 4.5; SEM = 0.04) and 46 to 60 (LS-mean = 4.7; SEM = 0.03; LS-mean = 4.6; SEM = 0.04). In particular, this formulation was significantly less familiar to both younger men (LS-mean = 4.0; SEM = 0.05) and women (LS-mean = 4.2; SEM = 0.05) than to men (31–45: LS-mean = 4.5; SEM = 0.06; 46–60: LS-mean = 4.6; SEM = 0.06) and women (31–45: LS-mean = 4.5; SEM = 0.05; 46–60: LS-mean = 4.5; SEM = 0.05) in other age groups.

### 3.3. JAR Data

**Figure 2:**
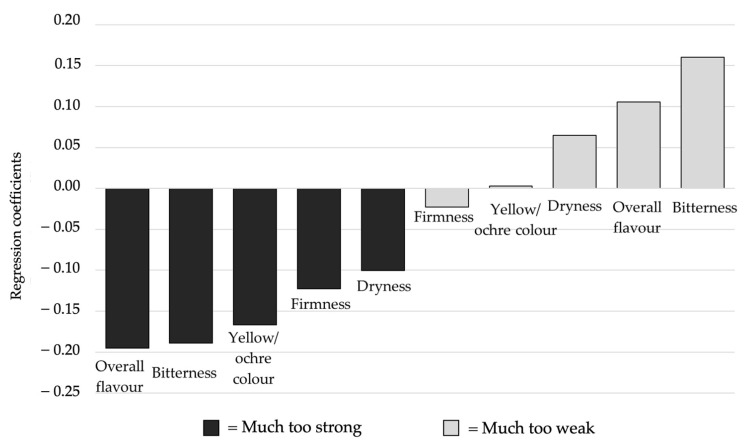
Regression coefficients obtained by the PLSR model relating overall liking and JAR data (*n* = 120) for the six samples.

The relationship between dummy variables and overall liking was investigated by PLSR modelling in order to establish which sensory attributes were mainly related to the samples liking. Regression coefficients calculated through PLSR modelling are depicted in Figure 2.

Liking was positively correlated with a low intensity of dryness, overall flavour, and bitterness, whereas a high intensity of overall flavour, bitterness, yellow/ochre colour, firmness, and dryness were associated with samples disliking.

### 3.4. Identification of Consumers’ Clusters

**Figure 3:**
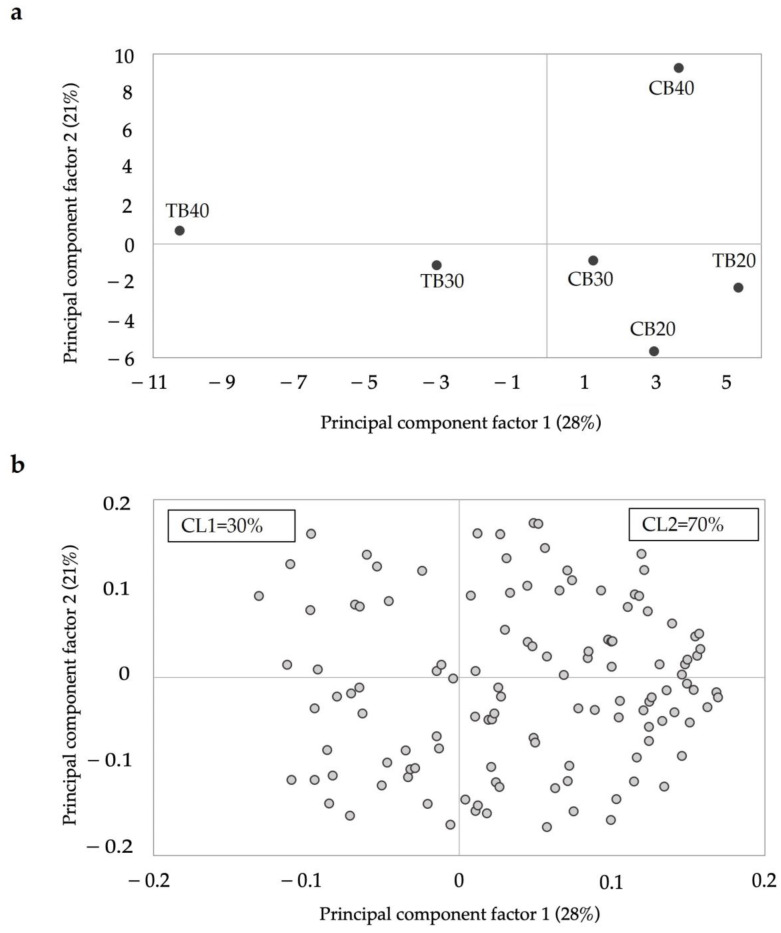
Principal component analysis (PCA) scores (**a**) and loadings (**b**) obtained from liking data of the six samples (CB = common buckwheat; TB = tartary buckwheat; CL1 = TB+_high likers; CL2 = TB+_low likers).

The scores and loadings plots obtained from the PCA model run on samples liking is reported in Figure 3a,b. The PCA explained 49% of the total variance, with PC1 accounting for 28% and PC2 contributing a further 21%. The scores plot shows that PC1 separates samples TB30 and TB40 (left panes of the plot) from the rest of the samples. Based on the PC1 of the loadings plot (Figure 3b), two clusters of consumers differing in liking were identified. We assumed that all subjects positioned in the negative part of PC1 (Figure 3b) were those preferring the formulations enriched with high percentages of tartary buckwheat (i.e., TB30 and TB40), while those in the positive part of PC1 preferred samples with common buckwheat (CB20, CB30, CB40) or a low percentage of tartary buckwheat (TB20). For the purpose of cluster identification, hereafter TB30 and TB40 are indicated as TB+. The PCA showed that 30% of consumers belonged to Cluster 1 (CL1: TB+_high likers = 30%) and the remaining 70% to Cluster 2 (CL2: TB+_low likers = 70%).

#### Characterisation of Consumers’ Clusters

**Figure 4:**
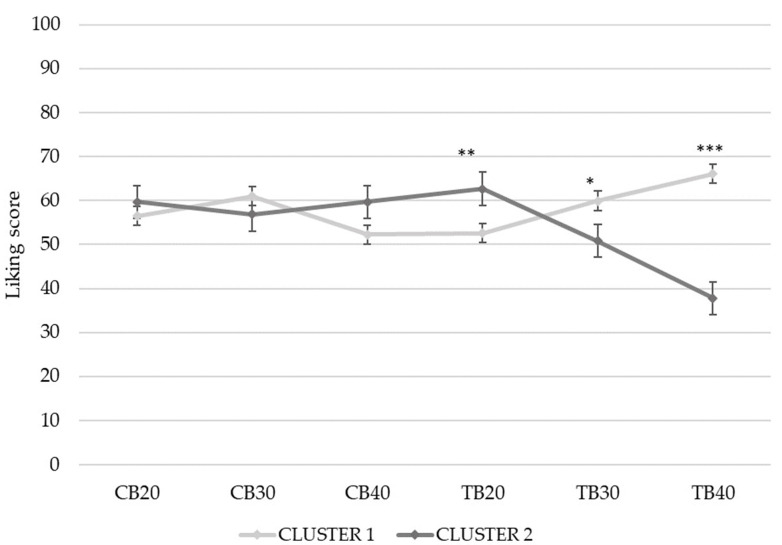
Mean liking score by samples (CB20, CB30, CB40, TB20, TB30, TB40) and clusters (Cluster 1: “TB+_high likers”; Cluster 2: “TB+_low likers”). Significant differences between clusters by sample are indicated by * *p* ≤ 0.05, ** *p* ≤ 0.01, and *** *p* ≤ 0.001. CB = common buckwheat; TB = tartary buckwheat.

The interaction cluster*samples showed a significant effect (F = 11.7; *p* < 0.0001) on liking scores. “TB+_high likers” represent those consumers which display an increased liking for the addition of tartary buckwheat, while “TB+_low likers” shows the opposite trend (Figure 4). The two clusters did not differ in liking for common buckwheat samples. Gender-related differences were found between the two clusters (χ^2^ = 9.3; *p* = 0.002). In “TB+_high likers”, men and women were comparable (*p* = 0.4), whereas in “TB+_low likers”, the proportion of women was higher than that of men (χ^2^ = 18.3; *p* < 0.001). No age-related differences were found between the two clusters.

**Figure 5:**
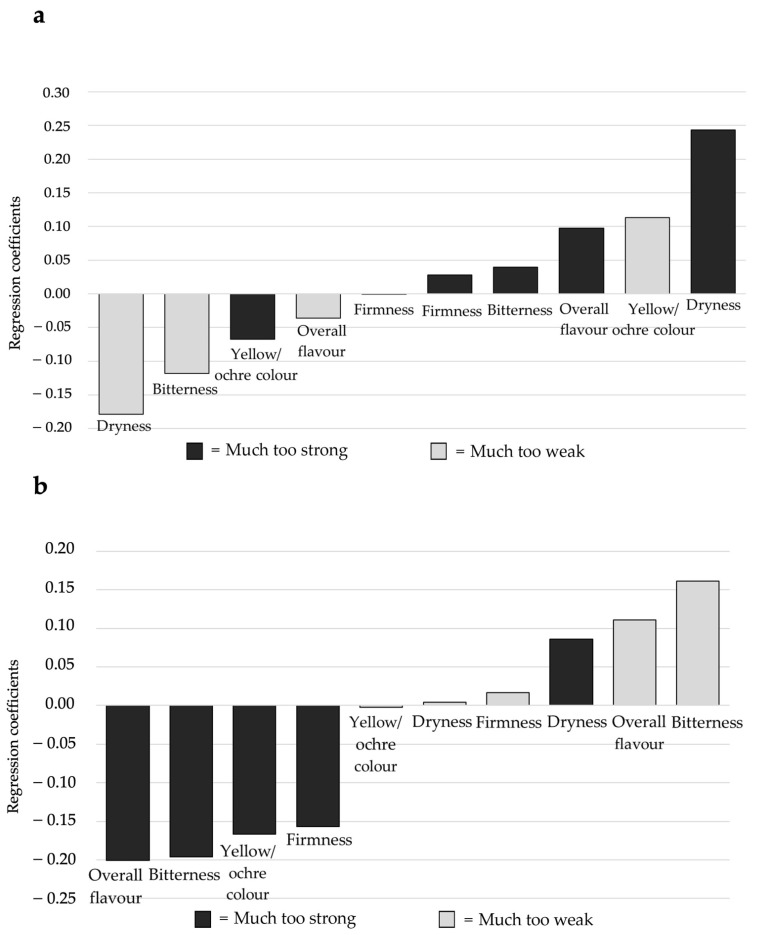
Regression coefficients of the PLSR model relating overall liking and JAR of “TB+_high likers” (*n* = 36) (**a**) and “TB+_low likers” (*n* = 84) (**b**) for each of the 10 dummy variables.

In order to explore which sensory attributes influenced the most samples liking in the two clusters, the PLSR model regression coefficients for “TB+_high likers” and “TB+_low likers” were calculated (Figure 5a,b). For “TB+_high likers” (Figure 5a), liking was positively correlated with an intense perception of dryness and overall flavour and a low intensity of ochre-yellow colour. On the other hand, a low intensity of dryness and bitterness were associated with samples disliking of this cluster. For “TB+_low likers” (Figure 5b), liking was mainly driven by a low intensity of the overall flavour and bitterness. A high perception of these descriptors as well as of yellow colour and firmness by “TB+_low likers” can be considered as a contributor to disliking.

Familiarity scores were found to be significantly different by cluster for corn-based formulation (*t* = −3.7; *p* < 0.0001). In fact, “TB+_low likers”, who preferred samples with low buckwheat additions, were found to be more familiar with corn-based formulations. In contrast, no significant differences between clusters were observed for liking scores for corn-based formulation enriched by the addition of buckwheat (*p* = 0.3). PROP responsiveness and food neophobia were not significantly different between clusters (PROP: *p* = 0.1; FNS: *p* = 0.8).

## 4. Discussion

Many studies have explored the technological, nutritional, and sensory properties of common buckwheat added in several food formulations [10,48,49]. Concerning tartary buckwheat, a few studies have dealt with its nutritional properties [5,8,14], while little knowledge exists about its sensory quality as well as the factors that may contribute to its liking and rejection.

The present study showed that corn-based gluten-free food formulations enriched with common buckwheat were accepted by consumers at all percentages of addition (up to 40%). The same trend was not found in samples containing tartary buckwheat since additions above 30% caused a decrease in hedonic appreciation. Higher additions of this pseudocereal produced changes in colour and overall flavour intensity, as well as in bitter taste and texture. In particular, the high intensity of these descriptors was identified as a driver of disliking. Since polyphenols are known to impart a bitter taste [50] and influence colour [51], it can be speculated that they may have influenced the colour and natural sweetness of the corn. In fact, bitter taste is considered a “warning sensation” that negatively influences food liking [29,37]. The fibre content of tartary buckwheat, besides contributing to the bitter taste, is also a possible cause of the darker colour [52], which can have a negative effect on liking. In fact, food colour is among the first sensory properties that are perceived by the consumer and can create a specific expectation in taste and flavour [53]. Furthermore, in this study, higher percentages of buckwheat addition were used in the corn-based formulations than those used on the market (around 20%). The higher concentrations of additions may have caused a darker colour to which the consumer is not accustomed.

Sensory expectations have been reported as extremely critical factors when selecting functional and/or sustainable foods over conventional ones [54]. In fact, consumers expect that the new product is as similar as possible to the conventional one from a sensory point of view [55,56]. Unfortunately, formulating foods with sustainable ingredients is a real challenge for the food industry since the addition of bioactive compounds or plant-based phytonutrients can cause bitterness, astringency, and/or off-flavours that compromise the sensory quality of the food, which consumers are not yet ready to give up in favour of a nutritional advantage [54]. For example, Stikic et al. [57] found that up to 20% additions of quinoa to a formulation for bread baking are accepted by consumers as they give the product a pleasant quinoa aroma. Instead, Demir et al. [58], using quinoa seed flour in pasta formulations, found that samples were acceptable up to 10% addition as higher concentrations caused a significant decrease in the liking due to the reduced brightness and yellowness of the pasta.

Another interesting result of the present study is that the samples enriched with buckwheat were particularly appreciated by consumers aged 46 to 60, who may represent a possible target of consumers of this type of product. The reason for this result can be explained by the fact that the food formulation based on corn flour and buckwheat, called “*polenta*” in Italy, represents a typical dish of the northern regions which is often eaten together with meat, cheese, or mushrooms. Currently, it is a well-known food product but may be consumed less frequently than in the past, when it was a very common dish due to being a very cheap and simple food. This hypothesis is also supported by the fact that corn-based formulation and corn-based formulation with the addition of buckwheat were found to be significantly less familiar to younger consumers aged 18 to 30 years. In fact, among the different factors influencing food acceptability, familiarity [59,60] and tradition play an important role, especially in older people [61].

In the present study, the identification of two clusters of consumers according to the hedonic evaluation revealed opposite behaviours with respect to tartary buckwheat samples. In fact, a group of consumers (30%) was found to like samples with high additions of this pseudocereal, so much so that liking increased as the percentage of addition increased. The two clusters varied in the perceived appropriateness of a series of sensory properties. In particular, it was shown that consumers’ liking of samples with high additions of tartary buckwheat was driven primarily by intense overall flavour and dryness and low intensity of yellow/ochre colour. However, characterisation of the two clusters showed no differences in responsiveness to PROP, which is a well-known marker of bitter perception and, in general, of taste and somatosensory sensitivity, or in gender, age, or behavioural attitudes. This result is interesting because it suggests that products with the addition of this pseudocereal have a good market potential if a suitable food matrix is chosen. In fact, corn-based flavour is naturally sweet and may contribute to masking the bitterness and strong overall flavour of this pseudocereal, encouraging consumption that allows for potential health benefits and important implications for biodiversity.

## 5. Conclusions

This study revealed that tartary buckwheat can be added to corn-based gluten-free formulations in concentrations up to 30% without compromising consumer acceptance. However, in 30% of consumers, high liking scores were associated with higher concentrations of tartary buckwheat. This indicates that there is a possible target of consumers who are willing to accept these products. One limitation of the study could be the choice of experimental food employed, which is a typical Italian product not widely consumed, except in some Italian regions. Further studies are needed to understand what kind of foods can be enriched with these flours, with the aim of producing new functional foods also for people with celiac disease.

## Figures and Tables

**Table 1:** Common (CB) and tartary buckwheat (TB) food samples.

Addition of Common or Tartary Buckwheat Flour (%)	Addition of Corn Flour (%)	Samples	
		CB	TB
20	80	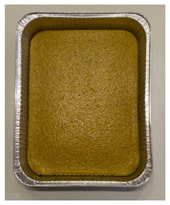	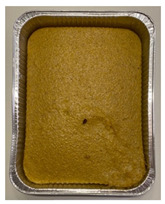
30	70	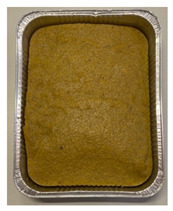	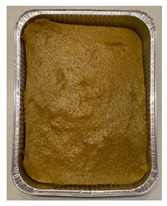
40	60